# Does pain take holidays? Non-attendance rates at a hospital-based pain clinic are elevated during the Jewish high-holidays

**DOI:** 10.1186/s13584-017-0132-9

**Published:** 2017-03-31

**Authors:** Motti Ratmansky, Nitzan Hai, Tzion Schlossberg, Aviva Mimouni-Bloch, Avraham Schweiger

**Affiliations:** 1 Pain Unit, Loewenstein Rehabilitation Hospital, 278 Ahuza Street, 43100 Raanana, Israel; 2 Sackler Faculty of Medicine, Tel Aviv University, Tel Aviv, Israel; 3 The Academic College of Tel Aviv, Tel Aviv, Israel; 4 Leumit health fund, Jerusalem, Israel

**Keywords:** Pain management, Pain clinic, Non-attendance, Gender differences

## Abstract

**Background:**

Patient non-attendance is an expensive and persistent problem worldwide with rates between 5–39% reported in the literature. The objective of the study was to assess whether there is a higher incidence of non-attendance in a hospital-based pain clinic during the period of the Jewish High Holidays (Rosh-Hashanah to Sukkot) and whether this is further compounded by other factors, such as demographic characteristics and previous visits to the clinic.

**Methods:**

Records were taken from the Lowenstein Rehabilitation Hospital appointment scheduling system. Data was gathered from two time-periods: High-Holidays and Control for each year, over a total of 6 years 2008–2013. Non-attendance was analyzed by period, by age, by gender and by previous visits to the clinic.

**Results:**

In the entire population studied (666 distinct records), the non-attendance rate was higher during the High-Holidays as compared to the Control period (32 vs. 24.1%; *p* = 0.030). Non-attendance rates were significantly higher during the Holidays among repeating patients (28.6 vs. 14.8%; *p* = 0.002) and among women (34.6 vs. 20.7%; *p* = 0.004).

**Discussion:**

Our data suggest that non-attendance is elevated during the High-Holidays in specific groups of patients, namely, repeating patients and women. Despite no direct inquiry into the reasons for non-attendance, we speculate that the elevated well-being and familial support during the holidays contribute to the patients’ ability to cope with persistent pain and possibly directly reduce the amount of pain, leading to patients missing their pain clinic appointments.

**Conclusion:**

Our results, provided they can be corroborated by larger-scale studies, can assist in scheduling policy adjustments such as avoidance of appointments during the High-holidays for specific patient populations and more rigorous reminder efforts during these times of the year that may lead to reduction in overall non-attendance rates in the pain clinic. Further, our data provide an impetus for further studies of non-attendance patterns among pain clinic patients, in order to acquire a better understanding of the reasons for non-attendance and develop strategies to reduce it and thus contribute to the continuous improvement of the Israeli health systems as well as others worldwide.

**Electronic supplementary material:**

The online version of this article (doi:10.1186/s13584-017-0132-9) contains supplementary material, which is available to authorized users.

## Background

The phenomenon of patients missing their appointments at medical clinics is well-documented. Studies on non-attendance rates report a fairly wide range of rates in clinics and health care centers in various medical fields. Among the findings are non-attendance rates of up to 12% in the United Kingdom, 5–55% in the United States, 4% in Denmark, 15% in Spain, and 27–36% in Israel [1–8].

This phenomenon results in a significant waste of time and money. In view of its economic consequences, and given a significant rate of non-attendance, most research in this area was carried out to improve the efficiency of the appointment system and to find economic solutions for reducing the phenomenon, such as different methods for providing notifications, reminders, and incentives to patients [5]. Some of the studies attempted to examine the reasons for patients not showing up for their appointments. Age, gender, socioeconomic status, accessibility of the clinic, the extent of the physician’s employment at the clinic, and the degree of familiarity with the physician are among the many and varied factors identified as having an impact on non-attendance rates [1, 2, 9–12]. Others focused on characterizing the profile of the population of patients who missed their appointments, depending on the specialization of the clinic, with reference to various parameters such as age, family status, severity of symptoms, substance abuse, and others [10, 13].

A different aspect of patient non-attendance is revealed by examining the phenomenon’s seasonal and monthly variation throughout the year, with specific focus on school vacations and holidays. This is a factor that can be addressed by the health care system, and may harbor a simple, cost-effective, and efficient solution: adjustment of appointment scheduling to reduce the overall frequency of non-attendance [3].

One study found a significant increase in non-attendance rates during the summer among patients treated at an allergy clinic [14]. Others found differences between certain days of the week [3].

At the same time, it appears that the psychosocial mechanisms that may explain why patients miss appointments made for dates falling on specific time periods such as holidays and half holidays (Hol Hamoed, in Israel) have not been thoroughly examined. A study looking at the effects of Christmas on the psychiatric population in the US showed that during this period there is a general decline in the use of psychiatric emergency services and a decrease in self-inflicted injuries and suicide attempts. The researchers concluded that Christmas has a protective effect with regard to many forms of psychopathology, except for increases in mood deterioration and cases of alcohol poisoning [15].

Another study examined the phenomenon of rebound, reflected by an increase in the number of patients seeking psychiatric services immediately after Christmas [16]. In contrast, studies have found that hospital visits due to chronic obstructive pulmonary disease exacerbations and cardiac events show peaks during Christmas [17–19].

Chronic pain is one of the most common health problems, affecting about 20% of the population [20, 21]. It can take the form of back pain, headaches and joint pain, among others, and it can severely diminish the patient’s physical and mental functioning [20]. There is a growing realization that persistent pain is a complex and multi-dimensional experience affected by biological, psychological, social, and spiritual factors [22]. According to the bio-psychosocial model of pain, which is now the most accepted heuristic approach to chronic pain [23, 24], various psychological states play a potential role in mediating the experience of pain [22]. In contrast to the emphasis placed by the biomedical model on the processes of disease, the integrated model regards the disease as an interaction between the various parameters, which shapes the person’s response to pain and affects one’s psychological and physical functioning [20, 24, 25].

Patient non-attendance is particularly relevant in the context of pain clinics that serve the community, mainly because of the long waiting-times prior to an appointment, exacerbated by the high degree of distress experienced by patients waiting for the appointment. To the best of our knowledge, no attempt has been made to examine the non-attendance rate of patients at pain clinics, and specifically not in the context of different periods throughout the year.

The Jewish High Holidays are a 3-week period, spanning the time from the Eve of the Jewish New Year (Rosh-Hashanah) until the end of Sukkot. Anecdotal evidence from pain specialists in Israel suggests that despite the suffering associated with chronic pain and the non-negligible waiting times, the non-attendance rate of pain clinic patients increases during the High Holidays.

The objective of the study was to examine whether there is a higher incidence of non-attendance in the pain clinic during the High Holidays, and whether this is further compounded by other factors, such as demographic characteristics and previous visits to the clinic. This analysis could potentially provide us with the profile of a patient who could be expected, with some degree of accuracy, to miss their High-Holiday appointment and allow us to consider appointment scheduling policies accordingly. In the long run, such adjustments may reduce the burden imparted by non-attendance and contribute to the ongoing improvement of the health system in Israel.

## Methods

### Population

Records were taken from the Lowenstein Rehabilitation Hospital appointment scheduling system. The study does not require ethical approval/statement.

Data were gathered from two time-periods: High-Holidays and Control periods for each year, over a total of 6 years 2008–2013. Exact dates of the holidays and Control periods are presented in Table 1.

**Table 1:** Date Ranges by Year

	2008	2009	2010	2011	2012	2013
Control	9/8–30/8	29/7–19/8	19/7–9/8	8/8–29/8	27/7–17/8	15/7–5/8
High-Holidays	30/9–21/10	19/9–10/10	9/9–30/9	29/9–20/10	17/9–8/10	5/9–26/9

The sample included all scheduled patients and divided into two groups: 247 patients scheduled for appointments at the clinic during the High Holidays and 419 patients with appointments scheduled during the Control period. Factors examined included: gender, age (all patients are adults; ages were grouped into categories: ≤44 years, 45–64 years and 65+) and whether this was the patient’s first visit or a repeat visit (a repeating patient was defined as a patient who had visited the clinic in the 3 years preceding the study).

Non-attendance was defined as an occurrence in which a patient had an appointment scheduled in the hospital’s scheduling system and had not arrived at the clinic for the appointment. Patients who had cancelled their appointment ahead of time were not included in the sample.

### Statistical analysis

Results were presented in contingency tables including counts and percentages. The proportion and 95% confidence intervals were calculated for each time period. Chi square test or Fisher exact test were used to compare the non-attendance rate for each sub group.

Analyses were carried out using IBM Corp. Released 2013. IBM SPSS Statistics for Windows, Version 22.0.0.1. Armonk, NY: IBM Corp figures were produced using R-project for statistical computing 3.1.2.

## Results

### Demographic characteristics

The study was based on 666 distinct records, 43.5% of them (290 records) first time visitors. Females comprised 55.6% of records; the youngest age group, ≤44 years, was the smallest (111 records, 16.8%), followed by 45–64 years (249 records, 37.6%) and 65 years or older (302 records, 45.6%). The raw data used in the analyses is provided in Additional file 1 Table S1.

### Non-attendance rate in the entire studied population

In the entire studied population for the combined study periods, the non-attendance rate was 27%. The non-attendance rate was higher during the High Holidays (32%) as compared to the Control period (24.1%) (*p* = 0.030, Fisher exact test; see Table 2).

**Table 2:** Non-Attendance Rates Summary Table

Measure	Level	Holidays				Control				Combined Periods				Non-attendance rate by period	Non-attendance rate by factor
		Arrived		Did not Arrive		Arrived		Did not Arrive		Arrived		Did not Arrive			
		*N*	%	*N*	%	*N*	%	*N*	%	*N*	%	*N*	%	*p*-value*	*p*-value**
Period		168	68.0%	79	32.0%	318	75.9%	101	24.1%	486	73.0%	180	27.0%	0.030	-
Age Group	18–44	27	67.5%	13	32.5%	48	67.6%	23	32.4%	75	67.6%	36	32.4%	1.000	<0.001
	45–64	53	55.8%	42	44.2%	107	69.5%	47	30.5%	160	64.3%	89	35.7%	0.030	
	65+	88	80.0%	22	20.0%	163	84.9%	29	15.1%	251	83.1%	51	16.9%	0.338	
Gender	Male	81	71.1%	33	28.9%	130	71.4%	52	28.6%	211	71.3%	85	28.7%	1.000	0.382
	Female	87	65.4%	46	34.6%	188	79.3%	49	20.7%	275	74.3%	95	25.7%	0.004	
Repeated Visit	No	68	63.6%	39	36.4%	117	63.9%	66	36.1%	185	63.8%	105	36.2%	1.000	<0.001
	Yes	100	71.4%	40	28.6%	201	85.2%	35	14.8%	301	80.1%	75	19.9%	0.002	

*p-value for the comparison between the Holiday and Control periods for each patient group

**p-value for the comparison between patient groups for non-attendance rate in the combined study periods

### Non-attendance rate among repeating patients

**Fig. 1:**
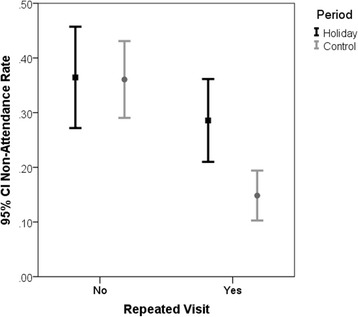
Ninety-five percent Confidence Interval for Non-Attendance Rate among Repeating and Non-Repeating Patients. Among repeating patients, the non-attendance rate was significantly higher during the High Holidays as compared to the Control period. In contrast, there was no difference in non-attendance rates between the High Holidays and Control periods among non-repeating (new) patients

In the combined study periods, the non-attendance rate among repeating patients (19.9% [95% CI:15.9%, 24.2%]) was significantly lower than among new patients (36.2% [95% CI:30.7%, 39.5%]) (*p* <0.001, Fisher’s exact test). Among repeating patients, the non-attendance rate was significantly higher (*p* = 0.002, Fisher exact test) during the High Holidays as compared to the Control period. In contrast, there was no difference in non-attendance rates between the High Holidays and Control periods among new patients (see Fig. 1).

### Non-attendance rate by gender

In the combined study periods, there were no significant differences in non-attendance rates between men (28.7% [95% CI: 23.6%, 32.6%]) and women (25.7% [95% CI:21.1%,29.4%]) *p* = 0.382.

**Fig. 2:**
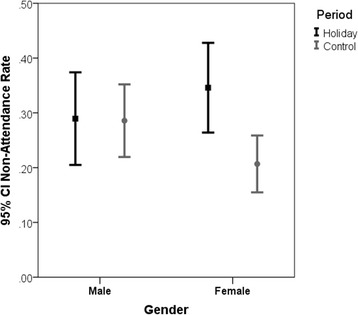
Ninety-five percent Confidence Interval for Non-Attendance Rate among Men and Women. Among women, the non-attendance rate was significantly higher during the High Holidays as compared to the Control period. In contrast, there was no difference in non-attendance rates between the High Holidays and Control periods among men

Among women, the non-attendance rate was significantly higher (*p* = 0.004, Fisher exact test) during the High Holidays as compared to the Control period. In contrast, there was no difference in non-attendance rates between the High Holidays and Control periods among men (see Fig. 2).

### Non-attendance rate by Age group

The non-attendance rate in the combined study periods was significantly lower (*p* <0.001, chi square test) among the eldest patients (65+; 16.9%) compared to the younger groups (35.7% for the group of 45–64 years old and 32.4% for the group of 18–44).

In the 45–64 years age group there was a significantly higher non-attendance rate during the High Holidays as compared to the Control period (44.2 vs. 30.5%, *p* = 0.030 Fisher’s exact test; see Table 2).

## Discussion

Patient non-attendance is an expensive and persistent problem worldwide, with rates between 5 and 39% reported in the literature [2]. Anecdotal evidence from pain specialists in Israel, suggesting that there is a peak in non-attendance in pain clinics during the High Holidays, has prompted the present systematic examination of this issue. This was further encouraged by the fact that there is a long, presumably distressful, wait for pain clinic appointments. We sought to establish this phenomenon, and improve understanding of the factors that affect non-attendance, thus leading to future solutions for reducing these rates.

In this study, we set out to find whether there was a difference in non-attendance rates in a hospital-based pain clinic during the High Holidays. We found that in the entire studied patient population, there was a statistically significant higher rate of non-attendance during the High Holidays. The difference was more pronounced among women and repeating patients.

The holiday period is characterized by social and family gatherings, leisure activities, a break from routine, and religious or spiritual engagement. It is possible that these provide a variety of opportunities for receiving social and instrumental support, distraction from the pain, and reduced focus on the body and ruminations about one’s distress. The holidays can also serve to distance individuals from everyday stressors and give them an opportunity to relax, reduce their anxiety, and improve their mood. These factors have been generally accepted as assisting in better handling of chronic pain and even in alleviating it [22, 23, 26, 27].

In our study, the group of repeating patients exhibited a lower non-attendance rate in the combined study periods compared to first time (new) patients. However, there was a significantly higher non-attendance rate during the High Holidays among repeating patients. Our study did not include questionnaires, and we did not ask patients to provide reasons for their non-attendance. We can only speculate that repeating patients generally keep their pain clinic appointments to maintain their long-term care. It is possible that elevated psychological well-being during the High Holidays is a factor in promoting the patient’s confidence that missing an appointment during the High Holidays will not irrevocably disrupt their long-term pain management. There was no inter-period difference in non-attendance rates among first time patients.

Some studies have found an elevated rate of non-attendance in women as compared to men [9], and others found the opposite [10]. Epidemiologic and clinical findings indicate that the burden of pain is substantially greater among women, and that women are more likely to seek health care for pain [28]. In the present sample of pain clinic patients, the proportion of women was 55.6%. In the combined study periods, there was no meaningful difference in attendance rates between men and women. However, there was a much higher, statistically significant, non-attendance rate among women during the High Holidays that was not observed in men. Other than the finding that the incidence of para-suicide cases was significantly lower during Christmas in women, but not in men [29], we did not find any studies in the literature that associated holidays and health complaints differentially between genders. As this was an archival study, we have no information regarding the reasons for non-attendance. The associations of affective variables with pain responses often differ for women as compared to men [28]. Thus, we can hypothesize that the positive psychological status and familial support during the holidays contribute to women’s ability to manage their pain without clinical intervention, more so than for men. Nevertheless, an alternative explanation could be women’s increased maternal and domestic task burden associated with the holiday period festivities. To assess this reasoning, such hypotheses should be studied further in the future using questionnaires enabling patients to provide reasons for their non-attendance.

In accordance with other studies, we found that non-attendance rates among the eldest patients were the lowest [9, 10, 12]. Among 45–64-year-olds, the non-attendance rate during the High Holidays was significantly higher as compared to the Control period. No meaningful inter-period difference was observed for the other age groups.

Our data suggest that non-attendance is elevated during the Jewish High Holidays, specifically among certain groups of patients, including repeating patients and women. The healthcare system and the patient have a shared interest in patients keeping their appointments, and thus behavior modification (“nudge”) methods, based on theories from the realm of behavioral sciences may be the key to reducing rates of non-attendance [30]. There is consistent evidence that reminder systems improve appointment attendance rates across a range of health care settings and patient population sub-groups [31, 32]. Different strategies to deal with non-attendance rates have been investigated, ranging from the more paternalistic approach of formulating a contract with the patient to attend future appointments [33] to the more relevant technological means of reminder systems such as phone calls, Short Message Service (SMS), Multimedia Messaging Service (MMS) and e-mails [31, 34–37].

“Simple” reminder massages, containing the appointment details (date, time, and location) were most frequently investigated and found to increase attendance rates compared to no-reminders. However, “reminder-plus” messages, that provide additional information (e.g. notification of appointment with a health promotional message or additional information regarding the medical procedures and the importance of follow-up) were found to be more effective than simple reminders at reducing non-attendance [31]. Text messaging reminders were found to be similar to telephone reminders in terms of their effect on attendance rates, and seeing as they cost less than telephone reminders, to be more-cost effective and to be superior in effect to postal reminders [32, 36]. Stating appointment costs in SMS reminders was found to further reduce non-attendance rates [37].

Based on our findings and the literature reviewed, we suggest simple strategies to increase attendance rates overall in the pain clinic and specifically during the High Holidays. Those can roughly divided into two broad mechanisms: behavior modification methods and policy adjustments.

The behavior modification methods can include the following:

1. Reminders: using the appropriate mode of technology (phone-based text messaging), preferably containing a “reminder-plus” message content tailored to specifically fit sub groups of patients for whom we have noted higher rates of non-attendance.
2. Populations in which high rates of non-attendance have been identified will get reminders via more than one mode of delivery (i.e. SMS, MMS and phone calls).
3. During the High Holidays, patients should be reminded multiple times, starting from a week before to a day before their appointment
4. Easy rescheduling: employing multiple systems for cancelling and rescheduling appointments such as automated SMS cancellation services, email services and other types of online scheduling systems.

Appointment scheduling policy adjustments could include the following:

1. Adjustment of clinic hours according to demand, by adding evening sessions to deliver more flexible hours to meet patients’ needs during the peak holiday period. This has been shown to be an effective strategy in a number of Family health clinics (Infant Care Centers) in Israel, have successfully raised attendance rates by adjusting their opening hours to meet the specific needs of the local working-mothers population.
2. Adjustment of appointment scheduling: avoiding scheduling appointments during peak holiday times and offering alternative dates before or after the holidays, this should be strongly suggested to women and to repeating patients.

Using the appropriate reminder strategy and the right scheduling policy adjustments can lead to a reduction of non-attendance rates in the pain clinic and the resource waste associated with it.

The pain clinic is an interesting test-case for a more general phenomenon of non-attendance, due to the high price (of physical distress and long waiting periods) endured by the patient. Our study was based on archival data; future studies would preferably include follow-up interviews and questionnaires with non-attenders to allow for better understanding of the reasons underlying non-attendance in general and specifically during the holidays. In the future, it would be interesting to further characterize the non-attending patients and more finely define specific groups of patients appropriate for further intervention, possibly combining data from other pain clinics.

## Conclusion

We found that among patients of a hospital-based pain clinic, there was a statistically significant higher rate of non-attendance during the High Holidays. The difference was more pronounced among women and repeating patients. Our opinion is that a combination of behavior modification methods and appointment scheduling policy adjustments can reduce the non-attendance rates.

We hope that this study will help in the promotion of a wide array of non-attendance studies in the Israeli health care system, providing data that will allow the informed formulation of solutions to this problem and contribute to the improvement of health systems in Israel and worldwide.

## Additional file

Additional file 1: Table S1. Data obtained from the hospital's appointment scheduling system. (XLSX 22 kb)
